# Integrated pulmonary index (IPI)-guided ventilation management during “tubeless” video-assisted thoracoscopic surgery under general anesthesia: a case report

**DOI:** 10.3389/fmed.2026.1722188

**Published:** 2026-02-02

**Authors:** Donglai Yan, Yu Bai, Yihua Li, Ling Yin, Yonghao Yu, Yang Yu

**Affiliations:** 1 Department of Anesthesia, Tianjin Medical University General Hospital, Tianjin, China; 2 Tianjin Institute of Anesthesiology, Tianjin, China

**Keywords:** integrated pulmonary index, respiratory monitoring, spontaneous ventilation anesthesia (SVA), tubeless anesthesia, video-assisted thoracoscopic surgery

## Abstract

**Background:**

“Tubeless” anesthesia for video-assisted thoracoscopic surgery (VATS) maintains spontaneous ventilation but complicates respiratory monitoring. The integrated pulmonary index (IPI) is a non-invasive tool that assesses respiratory status by integrating four real-time parameters [EtCO_2_, respiratory rate (RR), heart rate (HR), and SpO_2_] into a single score ranging from 1 to 10.

**Case presentation:**

We present a 42-years-old female who underwent right middle lobectomy under tubeless anesthesia with continuous IPI monitoring. Anesthesia was maintained via laryngeal mask airway with spontaneous ventilation, supplemented by total intravenous anesthesia and paravertebral blockade. The IPI system facilitated tiered respiratory management: IPI ≤ 6 signaled acute respiratory events, while IPI ≤ 4 necessitated immediate assisted ventilation intervention. Real-time IPI tracking ensured respiratory stability (target range 8–10, EtCO_2_ 35–45 mmHg) while promptly identifying deterioration. Postoperative arterial blood gases (PaCO_2_ 42 mmHg, pH 7.33) validated optimal ventilation management.

**Conclusion:**

This case demonstrates IPI’s efficacy in detecting early ventilatory compromise during tubeless VATS, enabling proactive intervention without compromising surgical advantages. Further refinement of IPI thresholds may enhance perioperative safety in spontaneous ventilation anesthesia.

## Background

“Tubeless” anesthesia for video-assisted thoracoscopic surgery (VATS) describes a technique that avoids tracheal intubation and typically maintains spontaneous ventilation as the primary mode of respiration (1). This approach falls under the broader paradigm of non-intubated thoracic surgery (NITS), whose principal aim is to circumvent the complications associated with endotracheal intubation and neuromuscular blockade. It is typically achieved by using a supraglottic airway device (e.g., a laryngeal mask airway), supplemented by regional anesthesia and intravenous sedation-analgesia. By minimizing the use of potent systemic anesthetics and avoiding mechanical ventilation, the tubeless approach has the potential to reduce postoperative discomfort and accelerate recovery (2, 3). This approach is not reserved only for patients with contraindications to endotracheal intubation, such as those with large bullae or severe respiratory impairment. In selected patients with good cardiopulmonary reserve, it may be intentionally chosen to minimize airway trauma, reduce postoperative discomfort, and potentially expedite recovery, provided that vigilant respiratory monitoring is maintained. However, the preservation of spontaneous breathing inherently carries a risk of respiratory impairment, such as hypoxemia and hypercapnia, thereby necessitating advanced and continuous respiratory monitoring throughout the procedure.

Traditional single-parameter monitoring, such as pulse oximetry, shows a delay in identifying respiratory impairment. Alarms are triggered only when hypoxia has already occurred, which means intensive intraoperative vital sign monitoring is essential (4). The integrated pulmonary index (IPI) is a non-invasive tool that assesses respiratory status by integrating four real-time parameters [EtCO_2_, respiratory rate (RR), heart rate (HR), and SpO_2_] into a single score ranging from 1 to 10 (4, 5). A score of 10 indicates normal respiratory function, while a score of 1 calls for urgent intervention (Table 1). Compared with conventional single-parameter monitoring, IPI has better clinical value in evaluating the adequacy of sedation. Its dynamic and real-time quantification of respiratory mechanics allows for highly specific and sensitive detection of ventilatory impairment, enabling preemptive intervention for respiratory complications (6).

**TABLE 1:** Clinical interpretation of the integrated pulmonary index (IPI).

IPI	Patient status subgroups	
10	Normal	Optimal values
8–9	With normal range	Suboptimal values
7	Close to normal range; requires attention	
5–6	Requires attention and may require intervention	
3–4	Requires intervention	
1–2	Requires immediate intervention	

This case report illustrates the clinical advantages of IPI monitoring in “tubeless” anesthesia, demonstrating its superior capability to maintain patient safety during spontaneous ventilation procedures. The findings highlight IPI’s potential to optimize respiratory monitoring and improve outcomes in VATS.

## Case report

A 42-years-old female [height, 158 cm; weight, 60 kg; BMI, 24.03 kg/m^2^; American Society of Anesthesiologists (ASA) physical status classification II; nonsmoker; no alcohol consumption] presented for evaluation of incidentally detected pulmonary nodules. Her medical history was unremarkable, with denial of hypertension, diabetes mellitus, coronary artery disease, or chronic pulmonary disorders. Preoperative pulmonary function tests revealed preserved spirometry (FEV1: 2.87 L, 112.7% predicted; FVC: 3.15 L, 105.9% predicted; FEV1/FVC: 91.30%) and a moderate reduction in diffusing capacity (DLCOc SB: 4.85 mmol/min/kPa, 60.3% predicted; DLCOc/VA: 1.10 mmol/min/kPa/L). Ventilatory reserve was adequate at 84.41%. Preoperative arterial blood gas analysis under room air showed pH 7.393, PaCO_2_ 39.46 mmHg, PaO2 92.8 mmHg, HCO_3_ 23.5 mmol/L, and SaO_2_ 97.97%, indicating normal baseline ventilation and gas exchange. High-resolution chest CT demonstrated: (1) a 9 mm × 6 mm subsolid nodule in the right middle lobe exhibiting pleural traction with indeterminate enhancement characteristics, and (2) a 5 mm ground-glass opacity in the left upper lingular segment. Additional findings included bilateral interstitial thickening and subcentimeter mediastinal lymph nodes in characteristic drainage stations (subcarinal, pretracheal, and aortopulmonary window regions). Cardiopulmonary architecture appeared otherwise unremarkable. These radiographic features were concerning for primary pulmonary malignancy, though inflammatory etiologies remained in the differential diagnosis. The patient was subsequently scheduled for thoracoscopic right middle lobectomy under general anesthesia.

Given her young age, ASA II status, preserved pulmonary function, and the goal of minimizing postoperative airway morbidity, a “tubeless” anesthetic technique using a laryngeal mask airway with spontaneous ventilation was selected, rather than conventional general anesthesia with a double-lumen tube. This decision was made after multidisciplinary evaluation, with a plan for continuous respiratory monitoring using the integrated pulmonary index (IPI) to ensure safety during spontaneous ventilation. Upon arrival in the operating room, standard intraoperative monitoring was established, including five-lead electrocardiography (ECG), continuous arterial blood pressure (ABP) monitoring via left radial artery catheterization, pulse oximetry (SpO_2_) and respiratory rate (RR) monitor. Peripheral venous access was established for the administration of lactated Ringer’s solution, and 0.3 mg of atropine was given to reduce secretions. Preoxygenation with 100% FiO_2_ was performed for 2 min prior to anesthesia induction. Induction was accomplished through sequential intravenous administration of 10 μg sufentanil, 10 mg remimazolam, 120 mg propofol. A size 4 laryngeal mask airway (LMA) was then successfully inserted, establishing the “tubeless” airway. To ensure stability during anesthesia induction and positioning, the patient was initially supported with synchronized intermittent mandatory ventilation (SIMV) mode with the following settings: tidal volume (VT): 480 mL, respiratory rate (RR): 13 breaths per minute, inspiratory-to-expiratory ratio (I:E): 1:2, flow trigger sensitivity: 2–5 L/min. Total intravenous anesthesia (TIVA) was maintained using propofol (2–5 mg/kg/h), and remimazolam (0.5–1 mg/kg/h), notably, no neuromuscular blocking agents were administered throughout the procedure. The patient was positioned in the left lateral decubitus position, and an ultrasound-guided paravertebral block was performed at the T4–T6 levels (3–4 mL of 0.5% ropivacaine per space) to provide targeted somatic analgesia for the planned thoracoscopic port sites. Following sterile preparation and draping, the patient gradually regained spontaneous breathing. The core “tubeless” phase of the procedure then commenced, during which the right middle lobectomy was successfully performed via a port at the 5th intercostal space in the anterior axillary line while the patient maintained spontaneous ventilation. Adequate surgical exposure was achieved by allowing the operative lung to collapse naturally under open pneumothorax, facilitated by the patient’s spontaneous breathing. Although conventional one-lung ventilation was not employed, a bronchial blocker and emergency intubation equipment were immediately available throughout the procedure as part of a pre-established safety protocol. Hemodynamic parameters remained stable throughout the procedure: heart rate 60–100 bpm, arterial blood pressure 120–140/50–65 mmHg, SpO_2_ 90%–100%, EtCO_2_ 30–50 mmHg and bispectral index (BIS) 40–60.

**FIGURE 1:**
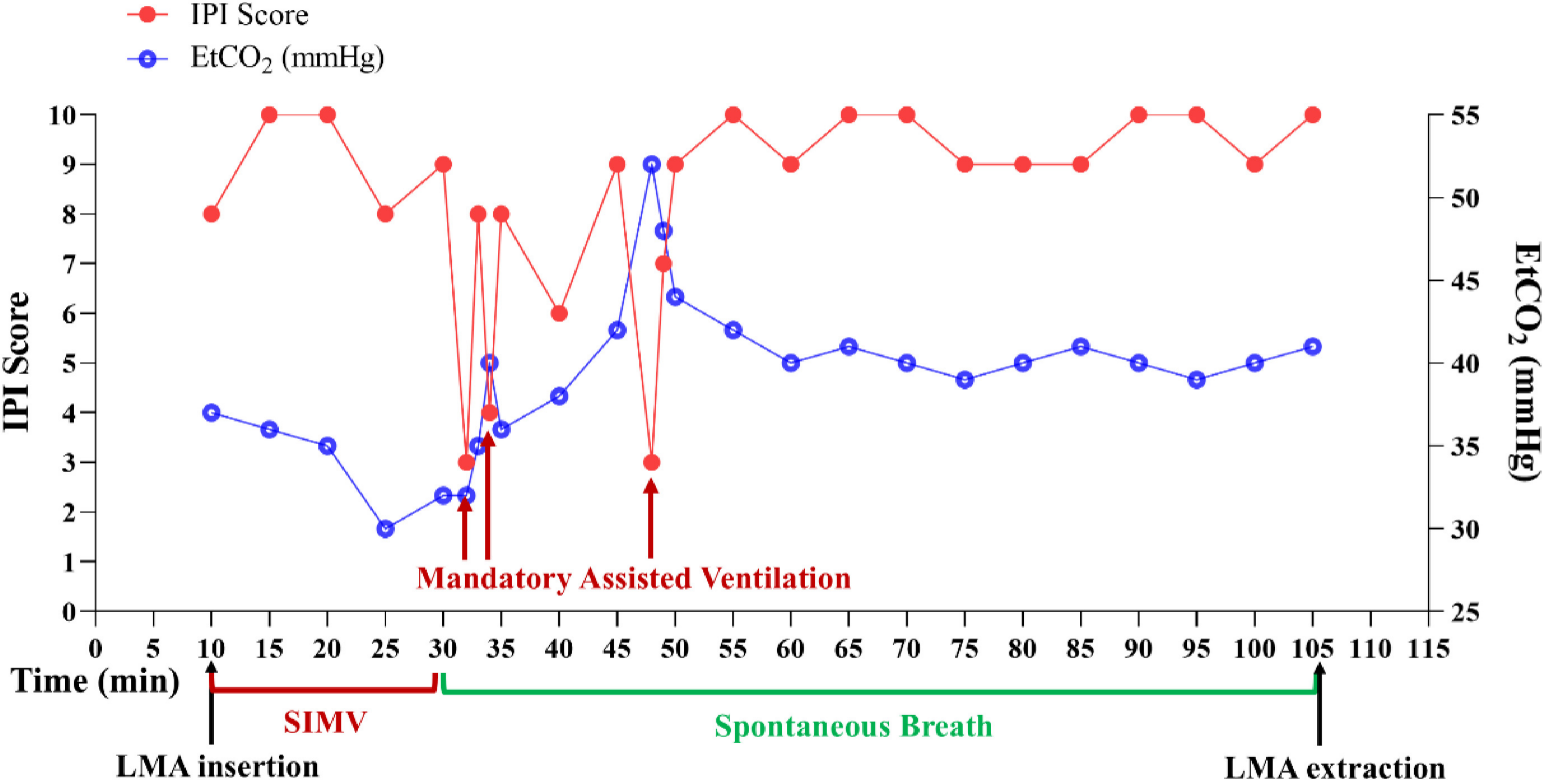
Trends of intraoperative IPI. Following LMA placement, continuous IPI monitoring was established to detect respiratory events. During SIMV ventilation, IPI was maintained at 8–10. For spontaneous ventilation: IPI ≤ 6 triggered monitoring alerts, while IPI ≤ 4 mandated immediate mandatory assisted ventilation to restore optimal IPI (8–10) and gas exchange, continued until stable spontaneous breathing resumed. IPI, integrated pulmonary index; EtCO_2_, end-tidal carbon dioxide; LMA, laryngeal mask airway; SIMV, synchronized intermittent mandatory ventilation.

Upon successful placement of the LMA, a multi-parameter respiratory function monitor (#JSD-JHY-001, Jinshengda Medical Supplies Co., Ltd., Hebei, China) was connected to enable immediate detection of IPI and other emergent respiratory events throughout the procedure. The IPI demonstrated characteristic patterns throughout the anesthetic phases. During the SIMV ventilation phase, the IPI was maintained between 8 and 10, indicating optimal respiratory and hemodynamic stability, confirmed adequate gas exchange and ventilatory efficiency. During the initial phase of spontaneous breathing recovery, the IPI fluctuated between 0 and 9, consistent with an EtCO_2_ of 40–52 mmHg (hypercapnia), HR 80–100 bpm, SpO_2_ 95%–100%, RR 0–21 times/min, indicating partial respiratory recovery but insufficient ventilation efficiency. An automated alarm was triggered when IPI values fell below 6, alerting the anesthesiologist to potential respiratory compromise despite SpO_2_ readings of 95%–100%. When the IPI ≤ 4, in response, manual mandatory assisted ventilation was provided to maintain adequate gas exchange (IPI ≥ 8). During the phase of complete spontaneous ventilation, the IPI stabilized at 8–10, aligning with an EtCO_2_ of 35–45 mmHg, HR 60–100 bpm, SpO_2_ 95%–100%, RR 15–20 times/min, which indicated appropriate ventilation settings and stable respiration. Intraoperatively, respiratory parameters remained stable with occasional adverse respiratory events (including respiratory depression, bradypnea, and end-tidal CO_2_ accumulation). The clinical protocol for IPI monitoring follows a tiered approach: When IPI values ≤6, the system triggers automated alerts to prompt close clinical observation without immediate intervention. However, if IPI ≤ 4, immediate mandatory assisted ventilation support is initiated to restore adequate gas exchange and maintain the IPI within the optimal target range of 8–10. This intervention continues until the patient’s spontaneous breathing is fully reestablished and stable within the desired parameters. The system maintains continuous monitoring throughout all phases to ensure prompt detection of respiratory changes (Figure 1). The procedure was completed uneventfully with an operative time of 1 h and 15 min and total anesthesia duration of 1 h and 55 min. Before the end of the surgery, 100 mg of flurbiprofen was intravenously infused for analgesia and 2 mg of tropisetron for antiemesis. Postoperative blood gas analysis demonstrated normal respiratory function without evidence of hypercapnia or acidosis (pH 7.33, PaCO_2_ 42 mmHg, PaO_2_ 143.75 mmHg).

Postoperatively, the patient was monitored in the PACU for 1 h with stable vital signs before being safely transferred to the ward. Her postoperative pain was well-controlled, with Numerical Rating Scale (NRS) scores of 2 at rest and 4 during coughing over the first 24 h. Notably, postoperative analgesia was achieved solely with intravenous flurbiprofen axetil (100 mg), with no opioid administration required throughout the entire postoperative course. The patient experienced no postoperative nausea or vomiting (PONV). She received supplemental oxygen via nasal cannula for 24 h postoperatively, maintaining oxygen saturation above 96%. Chest radiography prior to discharge on postoperative day 2 showed no evidence of atelectasis or pneumothorax. Standard postoperative care included anti-inflammatory, analgesic, and acid-suppressive therapies, regular dressing changes, and successful removal of the thoracic drainage tube. Postoperative pathology results confirmed the following: (1) pathological examination of the right middle lobe resection specimen revealed invasive adenocarcinoma (predominantly acinar subtype) without pleural invasion; no residual carcinoma was identified at the resection margin. Immunohistochemical staining showed positive expression of TTF-1, CK7, and Napsin A in tumor cells, with negative expression of P40; (2) No metastatic carcinoma was detected in station 7 lymph nodes (0/2).

The patient exhibited an uneventful postoperative recovery course, with no evidence of adverse reactions, pleural effusion, or pneumothorax. She was discharged on the 2nd postoperative day following clinical improvement.

## Discussion

“Tubeless” anesthesia in video-assisted thoracic surgery (VATS), which maintains spontaneous ventilation while minimizing anesthetic requirements, has obvious advantages in reducing postoperative discomfort. However, this technique inherently carries the risk of intraoperative respiratory complications, such as hypoxemia and hypercapnia, which make comprehensive patient monitoring more complex (3). In the presented case, the patient exhibited dynamic respiratory changes throughout the tubeless thoracoscopic procedure, progressing through stable, transitional, and recovery phases. IPI monitoring played a crucial role in tracking these physiological transitions.

Although arterial blood gas analysis remains the gold standard for diagnosing respiratory failure, its invasive nature, discontinuous measurement capability, and time delay between sampling and obtaining results limit its clinical application (7, 8). Early warning systems that can timely identify respiratory impairment are particularly valuable in intensive care units (ICUs), postoperative ward, and high-dependency units, especially in resource-limited settings. During surgery, the surgical drapes obstruct direct visualization of respiratory movements, necessitating reliance on monitoring systems to assess adequate spontaneous ventilation. Conventional single-parameter monitoring often fails to promptly detect ventilatory abnormalities. This indirect assessment method inevitably introduces a time lag in respiratory status evaluation. The IPI addresses these issues by non-invasively and high sensitively integrating four parameters (EtCO_2_, respiratory rate, heart rate, and SpO_2_) into a single numerical score (1–10). In this case, IPI showed significant clinical value: a stable score of 10 was associated with normal respiration (EtCO_2_ 35–45 mmHg, SpO_2_ 98%–100%), while fluctuations between 3 and 9 during the transition to spontaneous ventilation, as the depth of intravenous anesthesia was lightened, indicated respiratory impairment even though SpO_2_ remained normal. The IPI alarm prompted immediate manual ventilation, and respiratory stability was restored within 10 s, ensuring optimal surgical conditions (4, 6). Compared with conventional monitoring, IPI has three distinct advantages: (1) non-invasive continuous monitoring overcomes the latency of intermittent blood gas analysis; (2) multi-parameter integration improves the sensitivity in detecting hypercapnia compared to single SpO_2_ monitoring; (3) the simplified 1–10 scoring system allows for rapid clinical assessment, making it particularly suitable for tubeless anesthesia that requires efficient respiratory surveillance (9, 10). This case highlights the complementary roles of IPI and EtCO_2_ monitoring in guiding intervention. The initial assisted ventilation was triggered at IPI ≤ 4, driven by acute bradypnea before a significant EtCO_2_ rise, demonstrating IPI’s unique sensitivity to early respiratory drive depression despite normal SpO_2_. A subsequent intervention was guided by persistent hypercapnia (EtCO_2_ ∼50 mmHg). Thus, IPI provided an early warning of ventilatory pattern failure, while EtCO_2_ was essential for managing gas exchange adequacy. Their combined use enabled proactive management: the IPI alarm prompted intervention for bradypnea before hypoxemia developed, changing decision-making that might have been delayed if relying on SpO_2_ or EtCO_2_ trends alone.

While this case highlights the potential utility of IPI in our non-obese patient (BMI 24.03 kg/m^2^), its application must be viewed in the context of evidence and limitations. The IPI algorithm has been previously investigated and validated in settings such as procedural sedation, emergency department monitoring, and in patients with chronic obstructive pulmonary disease (5, 6, 11). However, its use in tubeless thoracic anesthesia represents a novel application, and specific validation in this population is still limited. The intervention thresholds utilized in this report (IPI ≤ 6 prompting alertness and IPI ≤ 4 mandating intervention) are derived from manufacturer recommendations and prior validation studies in sedated patients (11) and have not been rigorously calibrated for non-intubated VATS. Furthermore, as an integrated index, IPI carries a risk of false-positive alarms or false-negative reassurance. Therefore, it cannot replace arterial blood gas analysis or clinical judgment and should not be used in isolation. Successful implementation requires that IPI be interpreted as a sophisticated early warning system within the full clinical context, supported by a well-established airway emergency protocol and coordinated team readiness to manage potential respiratory impairment (6, 12).

Beyond the technical limitations, patient selection is paramount. Extending this approach to more challenging populations, such as obese patients (BMI ≥ 30kg/m^2^), warrants specific consideration. Obesity, often a relative contraindication due to worsened respiratory mechanics in the lateral position, increases the risk of hypoventilation and hypercapnia during spontaneous ventilation (13, 14). However, emerging data suggest feasibility in selected cases with expert management (13). In this case, continuous IPI monitoring could be particularly valuable. The synthesis of multiple parameters into a single score may provide an earlier warning of subtle ventilatory compromise–a critical advantage in obese patients where clinical signs can be elusive. This underscores the potential for technologies like IPI to refine patient selection and enhance safety, potentially broadening the applicability of “tubeless” strategies within a rigorous framework.

In conclusion, this case shows that IPI is an effective respiratory monitoring tool during tubeless anesthesia in VATS. Its early warning capability and real-time assessment help achieve an optimal balance between surgical benefits and respiratory risks. Future studies should further explore application thresholds to improve respiratory management in tubeless VATS.

## Data Availability

The original contributions presented in this study are included in this article/supplementary material, further inquiries can be directed to the corresponding author.
